# Computed-Tomography as First-line Diagnostic Procedure in Patients With Out-of-Hospital Cardiac Arrest

**DOI:** 10.3389/fcvm.2022.799446

**Published:** 2022-02-03

**Authors:** John Adel, Muharrem Akin, Vera Garcheva, Jens Vogel-Claussen, Johann Bauersachs, L. Christian Napp, Andreas Schäfer

**Affiliations:** ^1^Department of Cardiology and Angiology, Cardiac Arrest Centre, Hannover Medical School, Hannover, Germany; ^2^Department of Diagnostic and Interventional Radiology, Hannover Medical School, Hannover, Germany

**Keywords:** intensive care, computed tomography, out-of-hospital cardiac arrest, resuscitation, post-resuscitation treatment, return of spontaneous circulation

## Abstract

**Background:**

Mortality after out-of-hospital cardiac arrest (OHCA) with return of spontaneous circulation (ROSC) remains high despite numerous efforts to improve outcome. For patients with suspected coronary cause of arrest, coronary angiography is crucial. However, there are other causes and potentially life-threatening injuries related to cardiopulmonary resuscitation (CPR), which can be detected by routine computed tomography (CT).

**Materials and Methods:**

At Hannover Medical School, rapid coronary angiography and CT are performed in successfully resuscitated OHCA patients as a standard of care prior to admission to intensive care. We analyzed all patients who received CT following OHCA with ROSC over a three-year period.

**Results:**

There were 225 consecutive patients with return of spontaneous circulation following out-of-hospital cardiac arrest. Mean age was 64 ± 13 years, 75% were male. Of them, 174 (77%) had witnessed arrest, 145 (64%) received bystander CPR, and 123 (55%) had a primary shockable rhythm. Mean time to ROSC was 24 ± 20 min. There were no significant differences in CT pathologies in patients with or without ST-segment elevations in the initial ECG. Critical CT findings qualifying as a potential cause for cardiac arrest were intracranial bleeding (*N* = 6), aortic dissection (*N* = 5), pulmonary embolism (*N* = 17), pericardial tamponade (*N* = 3), and tension pneumothorax (*N* = 11). Other pathologies were regarded as consequences of CPR and relevant for further treatment: aspiration (*N* = 62), rib fractures (*N* = 161), sternal fractures (*N* = 50), spinal fractures (*N* = 11), hepatic bleeding (*N* = 12), and intra-abdominal air (*N* = 3).

**Conclusion:**

Early CT fallowing OHCA uncovers a high number of causes and consequences of OHCA and CPR. Those are relevant for post-arrest care and are frequently life-threatening, suggesting that CT can contribute to improving prognosis following OHCA.

## Introduction

Out-of-hospital cardiac arrest (OHCA) remains a striking challenge for emergency and intensive care medicine, and constitutes one of the main causes of in-hospital mortality worldwide (1). More than 50% of OHCA cases are caused by coronary ischemia (1–4). In Europe, mortality rates after OHCA are highly variable between countries (5, 6). Overall mortality and neurologic outcome are determined by the cause of arrest and its management. Awareness for basic life support in the general population is extremely heterogeneous despite broad education programs and telephone-guided resuscitation instructions (5). While algorithms and standards of care for immediate life support are constantly progressing, in-hospital post-resuscitation care is still less standardized (7). Coronary angiography and percutaneous intervention have emerged as a central part of early in-hospital care following OHCA due to the high prevalence of myocardial infarction as cause of arrest (3, 4, 7). Urgent coronary angiography is recommended by the European Resuscitation Council (ERC) and European Society of Cardiology guidelines in case of return of spontaneous circulation (ROSC) with ST-elevation and in resuscitated patients without ST-elevation, if myocardial infarction is assumed (8, 9).

In addition to invasive assessments, an increasing number of cardiac arrest centers employ whole body computed tomography (CT) into their standard of care (10–12). However, these advanced diagnostics are not yet fully implemented as immediate post-resuscitation care by current guidelines (7, 13). The 2021 ERC guidelines recommend CT only in case of suspected non-cardiac cause of arrest or when no coronary cause of arrest could be identified during coronary angiography (7).

The Hannover Cardiac Resuscitation Algorithm (HaCRA) includes routine coronary angiography and CT immediately after hospital admission for OHCA as a standard of clinical care (14). Here, we report whether CT scans as a standard diagnostic procedure provided important additional information and improved diagnostic accuracy.

## Materials and Methods

### Study Design

The HAnnover COoling REgistry -HACORE- is a prospective observational registry approved by the ethics committee at Hannover Medical School (#3567-2017) and is in accordance with the Declaration of Helsinki. HACORE includes all OHCA patients treated with therapeutic hypothermia at our institution (14).

### Patient Population

From HACORE we identified all patients admitted to our center receiving immediate (<2 h after admission) CT as part of the HaCRA standard during a continuous three-year period prior to the COVID-19 pandemic (14).

**Figure 1:**
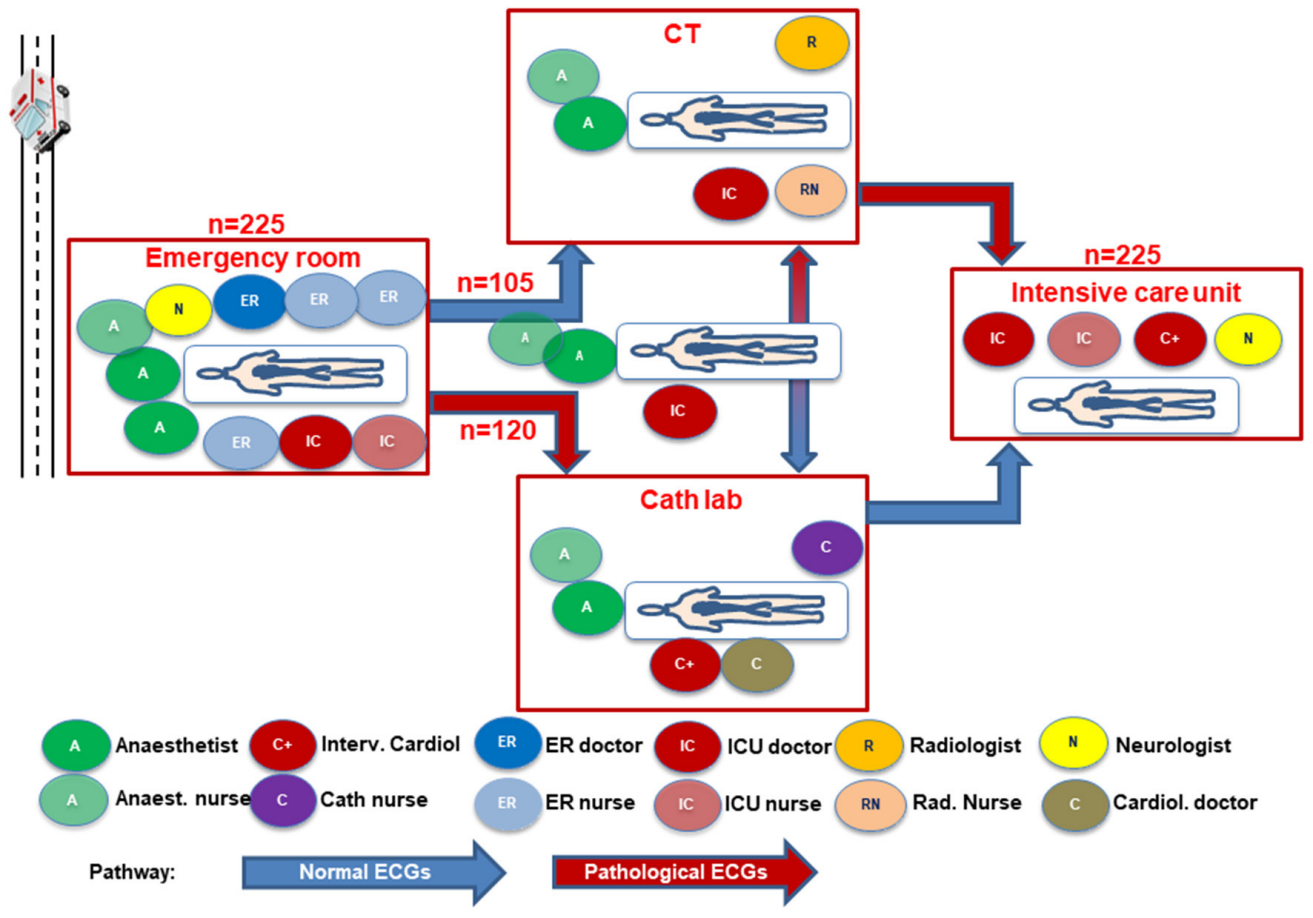
Intrahospital patient flow and human resource allocation for patients with ROSC after OHCA depending on a suspected primary coronary or non-coronary cause of arrest; CT – computed tomography; ER, emergency room; ICU, intensive care unit.

Patients included were aged >18 years, had non-traumatic OHCA with successful ROSC either prior to admission or in the emergency room, 12-lead ECG following ROSC, and successful application of the HaCRA algorithm (Figure 1). Patients with refractory hemodynamic instability after OHCA were provided with eCPR at admission. Patients requiring early eCPR are associated with a relatively worse outcome due to their initial instability (15). In order to avoid bias by this particular collective, they were excluded from the present study. An ECG after ROSC suggestive for a coronary cause of arrest was defined by the presence of either ST-segment elevation/ depression or a new onset bundle branch block according to current guidelines (7). Written consent was obtained retrospectively by next of kin or, if not present, by legally authorized officials.

### The Hannover Cardiac Resuscitation Algorithm

All patients were treated according to a local diagnostic and interventional standard procedure (14) (Supplementary Figure 1). In brief, the algorithm includes a multidisciplinary work-up of OHCA patients starting in the emergency room with immediate endotracheal airway management if not achieved before, continuation of mechanical CPR by an automated compression device in case of ongoing CPR, and early determination of cardiac function by transthoracic echocardiography in patients with ROSC. All patients without suspicion of obvious non-cardiac causes of arrest such as intracranial bleeding or massive pulmonary embolism were transferred to the catheterization laboratory. Diagnostic coronary angiography is performed irrespective of the presence or absence of ST-segment elevations in a 12-lead-ECG followed by revascularization of angiographically relevant coronary stenosis. In case of cardiogenic shock, complete revascularization was attempted at that time (16, 17) along with mechanical circulatory support prior to PCI (16, 18, 19). CT was performed after coronary angiography in case of a presumably cardiac cause of arrest or upfront in patients with a suspected non-cardiac cause of arrest (Figure 1). Thereafter, patients were transferred to the cardiac intensive care unit where therapeutic hypothermia was performed using intravascular cooling catheters (Coolguard Quattro^®^, ZOLL Medical, San Jose, CA, USA) for at least 24 h depending on the duration of ROSC or presence of anoxic cause of arrest. An active cooling device had been chosen to select and maintain a constant target temperature of 32°C during hypothermia followed by controlled rewarming (0.25°C per h) and maintained normothermia for at least another 72 h (14, 20). Transfer of the patient between catheterization laboratory, CT, and intensive care unit was supervised at all times by a team consisting of an anesthesiologist, intensive care physician, and an anesthetic nurse.

### CT Protocol

All CT-scans were conducted on a dual source dual energy CT (Somatom Force, Siemens Medical Systems, Erlangen, Germany). The CT protocol always included a native cranial CT, a native chest CT, and an arterial phase i.v. contrast enhanced chest CT with coverage of the abdomen and pelvis. For the native head CT the scan parameters were: 120 kV, 330 mAs, CareDose, rotation time 1 s, collimation 64 x 0.6 mm, 1 mm reconstructed slice thickness, pitch 0.8. For the chest, abdomen and pelvis CT the scan parameters were: 120 kV, 140 mAs, CareDose, rotation time 0.28 s, collimation 192 x 0.6 mm, 1 mm reconstructed slice thickness, pitch 2.5. For arterial phase imaging 80–100 mL of non-ionic contrast agent were injected intravenously at 5 mL/s. Bolus tracking was performed at the ascending aorta using a 250 HU trigger setting. Routinely, coronal and sagittal reconstructions were performed. A board-certified radiologist analyzed the images directly after the CT scans were performed and reported clinically relevant findings to the cardiologist in the cardiac arrest center responsible for the patient. Findings were classified as potentially life threatening in case of intracranial or intra-abdominal bleeding, free intra-abdominal air, ileus, type A aortic dissection, pericardial tamponade, central or bilateral occlusive pulmonary embolism, tension pneumothorax or pneumothorax with severely aggravated ventilation parameters with rapid improvement after chest tube insertion.

### Statistics

Quantitative data are presented as mean ± standard error of mean (SEM), median and interquartile range (IQR), and ranges depending on distribution. Data were compared using the Student's *t*-test for normally distributed data or the Mann-Whitney U test for nonparametric data. Deviations from a Gaussian distribution were tested by the Kolmogorov-Smirnov test. Spearman's rank correlation for nonparametric data was used to test univariate correlations. Qualitative data are presented as absolute and relative frequencies and compared using the chi-square test or the Fisher's exact test, as appropriate. Data were analyzed using SPSS version 26.0 for Windows (SPSS Inc. Chicago, IL, USA). A *P* < *0.05* was considered statistically significant.

## Results

### Baseline Characteristics

During the reported three-year period, 225 consecutive patients with non-traumatic OHCA and ROSC were treated per protocol and analyzed. Mean age was 64 ± 13 years and 170 (75%) patients were male. Initial rhythm after CPR was shockable in 55% (ventricular tachycardia or ventricular fibrillation, *N* = 123). Most patients (77%) had witnessed cardiac arrest and received immediate bystander CPR (64%). The average time for reaching stable ROSC was 24 ± 20 min. ECGs after ROSC were suggestive for myocardial ischemia in 53%, and baseline characteristics of those patients were not significantly different compared to patients with non-suggestive ECGs, except for initial rhythm. A shockable rhythm was more frequent in the ischemia-suggestive ECG group (64 vs. 44%, *P* = *0.002*), whereas the non-suggestive ECG group had higher numbers of asystole as initial rhythm (44 vs. 25%, *P* = *0.003*) (Table 1).

**Table 1:** Demographics and baseline characteristics of patients.

**Characteristics**	**Overall *N* = 225 (100)**	Pathologic ECGs^*^N = 120 (53)	Normal ECGs^**^N = 105 (47)	***P***
Male sex	170 (75)	95 (79)	75 (71)	0.178
Age (years)	64 ± 13	65 ± 13	64 ± 14	0.372
Body mass index (kg/m^2^)	27 ± 7	27 ± 6	28 ± 8	0.253
Circumstance on admission				
Bystander CPR	145 (64)	77 (64)	68 (65)	0.926
Initial rhythm				
Asystole	76 (34)	30 (25)	46 (44)	0.003
Pulseless electric activity	17 (8)	9 (<1)	8 (<1)	1.000
Ventricular tachycardia/ fibrillation	123 (55)	77 (64)	46 (44)	0.002
Other	9 (4)	4 (<1)	5 (<1)	0.737
ROSC (min)	24 ±20	24 ±19	24 ±22	0.984
Ongoing resuscitation on admission	23 (10)	8 (<1)	15 (14)	0.060
Clinical chemistry on admission				
Potassium (mmol/l)	4,4 ± 1,02	4,23 ± 1,21	4,54 ± 2,21	0.841
Creatininkinase (U/l)	290 ± 191	312 ± 150	151 ± 201	0.041
Lactate (mmol/l)	8,34 ± 3,51	8,12 ± 3,12	8,71 ± 3,11	0.974
Hs-Troponin (ng/l)	690 ± 603	720 ± 513	466 ± 422	0.052
pH	7,17 ± 0,48	7,16 ± 0,43	7,17 ± 0,51	1.000
Pre-existing illness/Risk factors				
Smoking	70 (31)	40 (33)	30 (29)	0.441
Arterial hypertension	125 (55)	71 (59)	54 (51)	0.244
Hyperlipidemia	67 (30)	41 (34)	26 (25)	0.124
Positive family history for CAD	15 (7)	7 (<1)	8 (<1)	0.605
Diabetes	50 (22)	27 (23)	23 (22)	0.915
Preexisting CAD	47 (21)	26 (22)	21 (20)	0.759
Preexisting PAD	16 (7)	6 (<1)	10 (10)	0.205
Atrial fibrillation	49 (22)	26 (22)	23 (22)	0.966
Previous cerebral event (Stroke/TIA)	32 (14)	15 (13)	17 (17)	0.429
Chronic kidney disease	32 (14)	18 (15)	14 (13)	0.721
Circumstance in ICU				
Hemodialysis	67 (30)	28 (23)	39(37)	0.754
MCS	14 (6)	9 (8)	5 (5)	0.207
Mortality (30d)	113 (50)	55 (45)	58 (55)	0.913

*Data are shown as mean ± standard deviation, otherwise (%) percentage of all patients with available data*.

* *Defined as new onset left bundle branch block, ST-elevation/-depression on admission*.

** *Defined as ECG without signs suggestive for acute/chronic ischemia*.

*CAD, coronary artery disease; CPR, cardio-pulmonary resuscitation; ECG, electrocardiogram; MCS, mechanic cardiac support; PAD, peripheral artery disease; ROSC, return of spontaneous circulation; TIA, transient ischemic attack*.

### Computed Tomography

All 225 patients received a CT of the head, chest, and abdomen/pelvis. In 120 patients (53%), CT scans were performed after coronary angiography and upfront in the remaining 105 patients. Table 2 shows the results of cranial CT, of abdominal/pelvic CT, and those of chest CT. In the cranial CT screening a total of 17 pathological findings were described in 12 patients without ECG signs suggestive of myocardial ischemia and in five patients with ischemia-suggestive ECG patterns. The further subdivision to intracerebral, subarachnoidal, subdural, or subgaleal bleeding showed no significant difference between the two ECG groups (Table 2). Abdominal CT revealed a total of 41 positive results within 28 cases with concomitant ECG signs suggestive of myocardial ischemia; only 13 cases had a non-pathological ECG, without significant differences between groups (Table 2). The highest number of pathological findings were made in chest CTs (Table 2). Overall, 438 pathologies were described with a nearly equal distribution between patients with ECGs being either suggestive or non-suggestive for coronary ischemia (*N* = 229 vs. 209).

**Table 2:** Pathological findings on Computed tomography.

**Findings**	**Overall *N* = 225**	Pathologic ECGs^*^N = 120	Normal ECGs^**^N = 105	***P***
Cranial Computed tomography				
Intracranial bleeding	1 (<1)	0 (0)	1 (1)	0.467
Subarachnoid bleeding	4 (2)	2 (2)	2 (2)	1.000
Subdural bleeding	1 (<1)	1 (1)	0 (0)	1.000
Subgaleal bleeding	8 (4)	2 (2)	6 (6)	0.150
Skull fracture	3 (1)	0 (0)	3 (3)	0.100
Abdominal Computed tomography				
Liver bleeding	12 (5)	8 (7)	4 (4)	0.388
Liver cirrhosis	8 (4)	6 (5)	2 (2)	0.289
Intra-abdominal air	3 (1)	2 (2)	1 (1)	0.600
Mesenteric stenosis	13 (6)	10 (8)	3 (3)	0.092
Ileus	3 (1)	0 (0)	3 (3)	0.100
Invagination	2 (<1)	2 (2)	0 (0)	0.500
Chest Computed tomography				
Aortic aneurysm				
Ascending Aorta	14 (6)	8 (7)	6 (6)	0.791
Abdominal Aorta	7 (3)	5 (4)	2 (2)	0.453
Aortic dissection				
Type A	2 (<1)	2 (2)	0 (0)	0.500
Type B	3 (1)	2 (2)	1 (1)	1.000
Pericardial tamponade	3 (1)	2 (2)	1 (1)	1.000
Aspiration	62 (28)	34 (28)	28 (27)	0.780
Pulmonary embolism^a^	17 (8)	7 (6)	10 (10)	0.322
Pulmonary edema	20 (9)	11 (9)	9 (9)	0.876
Pneumothorax^b^	11 (5)	2 (2)	9 (9)	0.026
Lung mass	12 (5)	5 (4)	7 (7)	0.554
Pleural effusion	65 (29)	33 (28)	32 (30)	0.623
Rip fracture	161 (72)	88 (73)	73 (69)	0.492
Sternal fracture	50 (22)	26 (22)	24 (23)	0.830
Spinal fracture	11 (5)	4 (3)	7 (7)	0.355

*Data are shown as (%) percentage of all patients with available data*.

* *Defined as new onset left bundle branch block, ST-elevation/-depression on admission*.

** *Defined as ECG without signs suggestive for acute/chronic ischemia*.

a *Central or bilateral occluding embolism*.

b *Tension pneumothorax or aggravated ventilation parameters (↑pressure, ↓ tidal volume) with rapid improvement after chest tube insertion*.

**Figure 2:**
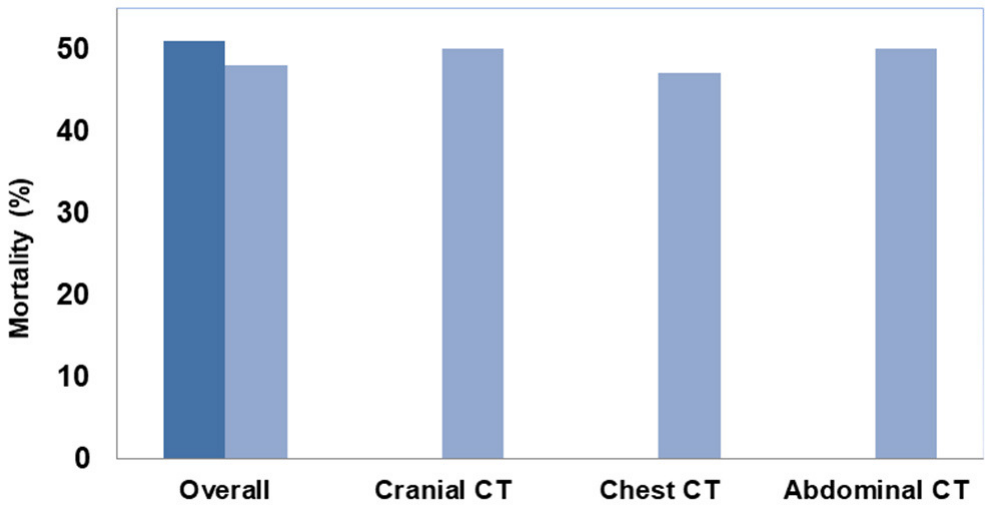
In-hospital mortality (%) in patients with no or non-critical (dark blue) compared to critical CT-findings (light blue) including separate evaluation of critical findings with respect to the imaging area.

Initially moderate pericardial effusions seen on echocardiography in the emergency room showed hemodynamic progression into pericardial tamponade in three cases as detected by CT-scan, of whom two had an ischemia-suggestive ECG. Furthermore, 17 patients suffered from central or bilateral occluding pulmonary embolism, 11 patients had pneumothoraxes, mostly represented in the patient group with ECGs non-suggestive for ischemia (*N* = 9). All of these patients showed aggravated ventilation parameters such as increased ventilatory pressures and decreased tidal volumes. One presented with a mediastinal shift and signs of tension pneumothorax that required immediate treatment. Bone fractures were located in mainly three areas: Sternal (*N* = 50), rib (*N* = 161), and spinal fractures (*N* = 11). Similarly, there was an equal distribution of fractures between groups with and without an ischemic ECG. Of all CT findings, 60 cases (27% of the total population) provided clinically relevant results, 28 (12%) in patients with ischemia-suggestive and 32 (14%) in patients with normal ECGs. Likely due to early CT diagnostics and the resulting initiation of chest drain insertions in case of pneumothorax or surgical treatment in case of aortic dissection, mortality was not increased compared to the remaining OHCA population. Overall, 113 patients (50%) died during the hospital stay. Of the 60 patients with critical CT-findings, 29 (48%) died, while 84 of the remaining 165 patients without critical pathologies in CT died (51%) (Figure 2). Therefore, rapid identification of potentially lethal comorbidities and subsequent causal treatment likely prevented increased mortality in this highly vulnerable subgroup [mortality 48 vs. 51%, *P* = 0.7646; relative risk 0.95 (95%-CI 0.70–1.28)].

Since prolonged time to ROSC may lead to more side effects and increased mortality, we examined whether time to ROSC was correlated with either mortality or pathological CT findings. Time to ROSC was shorter in survivors than in non-survivors (20.1 ± 1.3 min vs. 28.2 ± 2.3 min; *p* = 0.003). For most pathological CT findings there were no significant differences in time to ROSC except for patients with type A aortic dissection (65.1 ± 40.9 min vs. 23.2 ± 1.3 min; *p* = 0.004) or pericardial effusion (17.1 ± 3.4 min vs. 24.3±1.3 min; p = 0.037). We observed higher survival in patients with initial shockable rhythms compared to patients with non-shockable rhythms (65 vs 31%; *p* < 0.001). However, there was no significant difference in pathological CT findings with respect to the form of initial rhythm.

## Discussion

In this study on 225 patients with successful resuscitation after OHCA, CT on admission uncovered a substantial number of pathological and potentially life-threatening CT findings. Those were similarly distributed between patients with and those without ischemia-suggestive ECG patterns indicating a probable coronary cause of arrest. Rapid identification of potentially life-threatening conditions with subsequent intervention likely contributed to a comparable mortality compared to patients without such findings.

Results from *Coronary Angiography after Cardiac Arrest without ST-Segment Elevation (COACT)* and *Angiography after Out-of-Hospital Cardiac Arrest without ST-Segment Elevation (TOMAHAWK*) suggest that if no ST-segment elevation is present, delayed coronary angiography has no disadvantage for patients (21, 22). However, in the HACORE population we could recently show that the rate of culprit coronary stenosis was high even in patients without ST-segment elevation in first ECG after ROSC if there was an initial shockable rhythm.

There, early angiography was associated with similar favorable neurological outcome as in OHCA patients with ST-elevations (23). As supported by the trial results from COACT and TOMAHAWK and the real-world experience from HACORE, we do believe that a CT scan as initial diagnostic test should be favored in all OHCA patients without ST-segment elevations. Nevertheless, we feel that a CT does also provide valuable information in patients with ST-elevations, but the exam should be performed after successful revascularization and prior to ICU admission.

**Figure 3:**
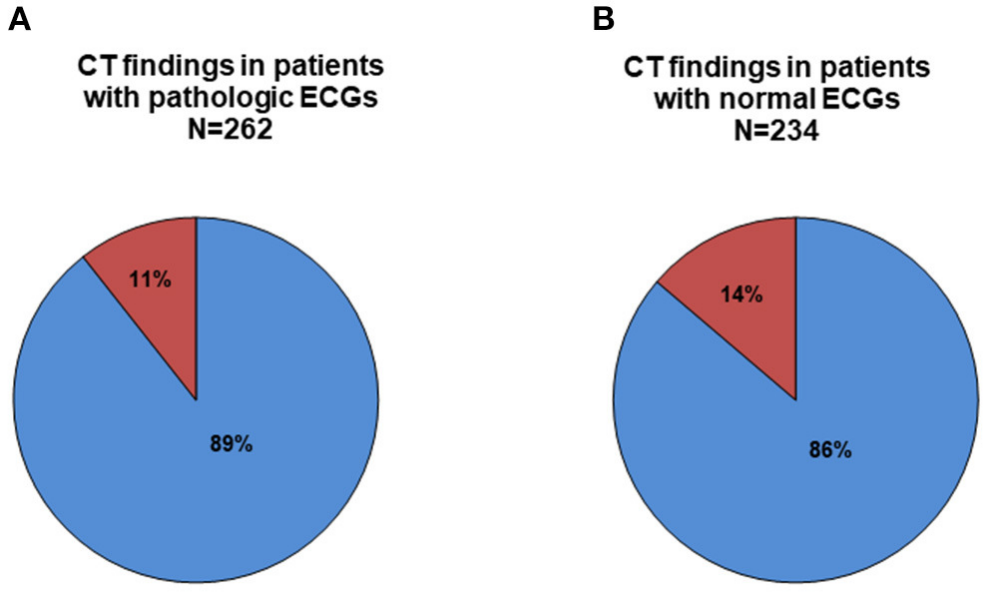
Proportion of patients (%) with critical (red) compared to non-critical (blue) CT-findings according to the electrocardiogram (ECG) being either suggestive for coronary ischemia **(A)** or not **(B)**.

A study from the *Parisian Region out of Hospital cardiac Arrest registry (PROCAT)* found that immediate coronary angiography irrespective of ECG findings would lead to an improved outcome and hospital survival (24). This was attributed to the fact that coronary artery disease and myocardial ischemia are the most common reason for OHCA (25). However, data from the same *PROCAT* registry also demonstrated that in addition to coronary angiography, CT would be the most effective diagnostic tool to detect any other OHCA etiology. The authors concluded that only a combination between those two diagnostic modalities would lead to a clear understanding of the particular case (10). Furthermore, the presence of comorbidities on CT was associated with in-hospital mortality (10, 26), most probably due to unmasking otherwise unseen extra-coronary pathologies. Accordingly, underdiagnosing comorbidities or resuscitation-related injuries would increase morbidity and mortality, counteracting all efforts of post-resuscitation care. Missing the frequent resuscitation-related injuries might significantly affect the outcome, if they remain undetected under routine intensive care (27). In turn, recognizing those patterns may translate into better outcome or at least prevent unintended complications such as persistent bleeding, e.g., caused by intensified anti-thrombotic medications following percutaneous coronary interventions (28, 29). The observation of 438 overall pathological findings in chest CTs from 225 patients in our registry is well in line with other studies focusing on complications resulting from CPR (27, 29–32). However, even the 2020 American Heart Association (AHA) Guidelines for Cardiopulmonary Resuscitation and Emergency Cardiovascular Care mention chest radiography instead of chest CT as the essential diagnostic test for intubated patients after OHCA (25). In contrast, CT shows a clear advantage compared to simple chest radiography, since it allows higher quality imaging and multiplane anatomical reconstruction (33). Chest radiography underestimates life-threatening complications such as pneumothorax, especially in supine position single-plane images (12, 33, 34). In addition, CPR-related rib and sternal fractures are underdiagnosed by conventional chest X-ray (35). This could translate into a more complicated management of ventilation leading to ventilator-associated lung injury and worsening overall outcome. CT currently represents the only gold standard for diagnosis or rule-out of certain life-threatening pathologies such as pulmonary embolism, aortic dissection, or intracranial bleeding (12). No other available diagnostic measure can provide rapid and definite diagnosis of potentially life-threatening pathologies in a single procedure. Measures such as sonography, echocardiography, or clinical examination are important diagnostic tools, but remain subjective, time-consuming, and insufficiently sensitive. In contrast, CT offers high quality multiplane imaging and the ability to record a whole body or larger area image in one setting. In many cases of OHCA a number of extra-cardiac pathologies detected by CT implicate an immediate threat and require adaptations of intensive care strategies in order to prevent deterioration. For instance, in cases of extracorporeal cardiopulmonary life support after OHCA, computed tomography provides sensitive information for further intensive care therapy. In a recent report in eCPR patients, routine CT diagnostic revealed a large number of relevant pathological findings similar to our observation in a broader patient cohort (36). During implementation of the 2010 American Heart Association Guidelines only 2.8% of detected pathologies were identified as directly life threating (37). However, every single cardiac arrest case has to be evaluated individually as circumstances of arrest and resuscitation differ widely. In this context, CT performed on admission provides essential information by ruling out immediately compromising conditions. Especially when considering the neurologic status after prolonged cardiopulmonary arrest in comatose patients, CT imaging helps to detect significant and irreversible pathologies on admission and may guide prognostication (28, 38). Therefore, CT imaging to detect brain injury and predict neurological outcome is, together with magnetic resonance imaging, the most studied neuroimaging modality (25). What distinguishes CT imaging from magnetic resonance imaging in the case of OHCA patients is its speed and advantageous ability in delivering additional information about structural lesions such as skull fractures. Using a radiological assessment of almost the whole body, early risk stratification for further invasive management in the intensive care setting can be planned (39). A positive finding in coronary angiography commonly represents a treatable cause of arrest and thus beneficially affects survival, also by reducing the likelihood of recurrent arrest. A “positive” finding on cranial CT scans is often associated with underlying severe damage of the brain and is most probably associated with worse outcome (10). The European Guidelines for Resuscitation Care recommend head- and thoracic- CT scans prior to ICU admission after OHCA (7). In our analysis, CT detected 496 findings with a nearly equal distribution in both pathological and normal ECG groups (*N* = 262 vs. 234) (Figure 3). Even in patients with ischemic ECGs and primary PCI concomitant extra-cardiac pathologies have to be anticipated and frequently require acute treatment. Only when combining coronary angiography with whole-body CT prior to ICU admission a complete diagnostic image of the patient after OHCA will be gained. Patient recruitment for the current analysis was before the COVID-19 pandemic. Even in that non-COVID context, early CT diagnostic during initial work-up revealed important information for treatment from thoracic pathologies (40). Taking the approaching fourth wave of the pandemic into account, the likelihood of false-positive rapid PCR or MDA tests is increasing. The extremely helpful ability of chest CT to indicate a pulmonary COVID-like pattern will provide useful additive information gained from post-arrest CTs, not only influencing care of the individual patient but also impacting on strategic allocation of patients within hospitals regarding a probable or non-probable COVID-19 disease (41).

### Limitations

This analysis comprises certain limitations. First, it was a retrospective single center observation. Second, it did not include other diagnostic measures that were performed immediately after admission such as echocardiography or abdominal ultrasound. Those potentially could detect pathologies or injuries secondary to CPR, either leading to a faster adjustment of management or, by missing such pathologies, emphasize the value of additional whole-body CT.

## Conclusions

Immediate whole-body CT in OHCA patients provides valuable information about non-coronary causes of cardiac arrest and resuscitation-related injuries affecting further treatment. If coronary angiography is primarily used based on a suspected coronary cause of arrest, a CT should be performed afterwards. Detection of potentially life-threatening comorbidities and their causal treatment is associated with comparable mortality as in patients without life-threatening complications. Therefore, CT should be routinely included in diagnostic workup of OHCA, irrespective of the presence or absence of ischemic ECG patterns.

## Data Availability Statement

The raw data supporting the conclusions of this article will be made available by the authors, without undue reservation.

## Ethics Statement

HACORE is a prospective observational registry approved by the Ethics Committee at Hannover Medical School (#3567-2017) and is in accordance with the Declaration of Helsinki.

## Author Contributions

AS, MA, and JB designed the registry. AS, MA, VG, LN, and JA recruited the patients and analyzed the data. AS, MA, and JA drafted the manuscript. VG, JV-C, LN, and JB critically revised the manuscript. All authors contributed to the article and approved the submitted version.

## Funding

This study was partly supported by the Clinical Research Group (KFO) 311 of the Deutsche Forschungsgemeinschaft (DFG).

## Conflict of Interest

AS received modest lecture fees from Zoll regarding therapeutic hypothermia. LN received lecture/proctoring/consulting honoraria and research funding from Abiomed, and lecture honoraria from Abbott, Maquet, Orion, and Zoll. The remaining authors declare that the research was conducted in the absence of any commercial or financial relationships that could be construed as a potential conflict of interest.

## Publisher's Note

All claims expressed in this article are solely those of the authors and do not necessarily represent those of their affiliated organizations, or those of the publisher, the editors and the reviewers. Any product that may be evaluated in this article, or claim that may be made by its manufacturer, is not guaranteed or endorsed by the publisher.

## Supplementary Material

Supplementary Figure 1 HaCRA work-flow for patients with out-of-hospital cardiac arrest or cardiogenic shock.

## Abbreviations

ACLS: Advanced cardiac life support
BLS: Basic life support
CPR: Cardiopulmonary resuscitation
CT: Computer tomography
ECG: Electrocardiogram
eCPR: extracorporeal cardiopulmonary resuscitation
HaCRA: Hannover Cardiac Resuscitation Algorithm
HACORE: Hannover Cooling Registry
ICU: Intensive care unit
LBBB: Left bundle branch block
MCS: Mechanic cardiac support
OHCA: Out- of- hospital cardiac arrest
PCI: Percutaneous coronary intervention
ROSC: Return of spontaneous circulation
STEMI: ST-elevation myocardial infraction.
