# Supplementary figures and images for: Computed-Tomography as First-line Diagnostic Procedure in Patients With Out-of-Hospital Cardiac Arrest
Source: Front Cardiovasc Med. 2022 Feb 3;9:799446. doi: 10.3389/fcvm.2022.799446 (PMC8850697; doi:10.3389/fcvm.2022.799446)

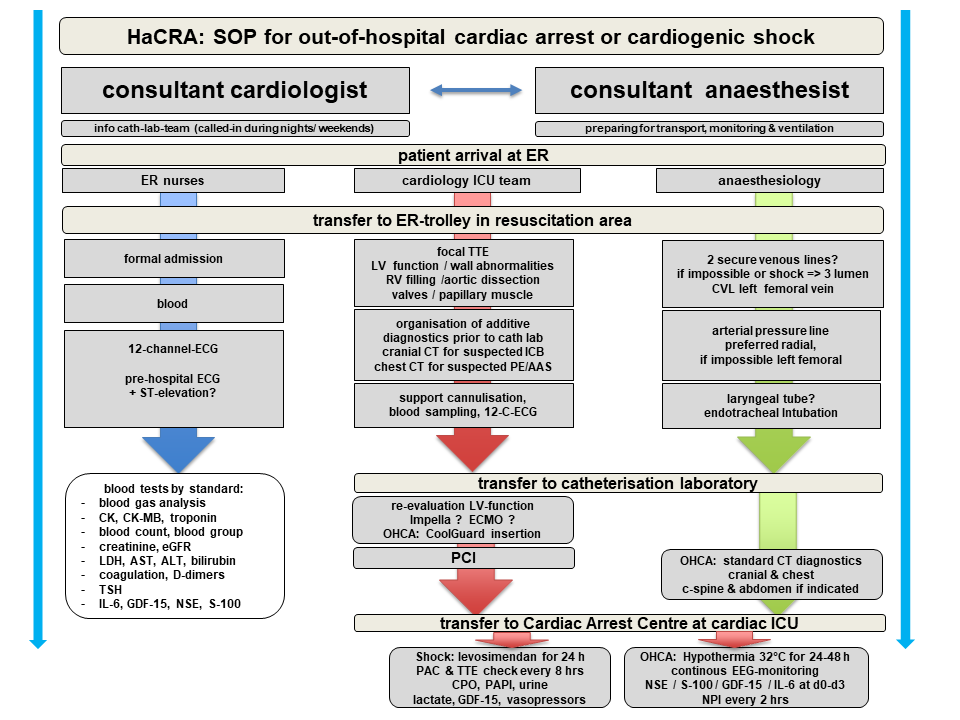

Supplement: Supplementary Figure 1 — HaCRA work-flow for patients with out-of-hospital cardiac arrest or cardiogenic shock. [file Image_1.TIF]
